# Laparoscopic One-Anastomosis Gastric Bypass: Technique, Results, and Long-Term Follow-Up in 1200 Patients

**DOI:** 10.1007/s11695-016-2428-1

**Published:** 2016-10-25

**Authors:** Miguel A. Carbajo, Enrique Luque-de-León, José M. Jiménez, Javier Ortiz-de-Solórzano, Manuel Pérez-Miranda, María J. Castro-Alija

**Affiliations:** Centre of Excellence for the Study and Treatment of Obesity and Diabetes, Calle Estacion, No. 12, 1°, 47004 Valladolid, Spain

**Keywords:** Morbid obesity, Bariatric surgery, Metabolic surgery, Laparoscopic gastric bypass, One anastomosis gastric bypass, OAGB, Single-loop gastric bypass, Mini-gastric bypass, MGB, Single-anastomosis gastric bypass, Billroth II gastric bypass, Omega loop gastric bypass, Weight loss, Diabetes surgery, Long-term follow-up

## Abstract

**Background:**

Excellent results have been reported with mini-gastric bypass. We adopted and modified the one-anastomosis gastric bypass (OAGB) concept. Herein is our approach, results, and long-term follow-up (FU).

**Methods:**

Initial 1200 patients submitted to laparoscopic OAGB between 2002 and 2008 were analyzed after a 6–12-year FU. Mean age was 43 years (12–74) and body mass index (BMI) 46 kg/m^2^ (33–86). There were 697 (58 %) without previous or simultaneous abdominal operations, 273 (23 %) with previous, 203 (17 %) with simultaneous, and 27 (2 %) performed as revisions.

**Results:**

Mean operating time (min) was as follows: (a) primary procedure, 86 (45–180); (b) with other operations, 112 (95–230); and (c) revisions, 180 (130–240). Intraoperative complications led to 4 (0.3 %) conversions. Complications prompted operations in 16 (1.3 %) and were solved conservatively in 12 (1 %). Long-term complications occurred in 12 (1 %). There were 2 (0.16 %) deaths. Thirty-day and late readmission rates were 0.8 and 1 %. Cumulative FU was 87 and 70 % at 6 and 12 years. The highest mean percent excess weight loss was 88 % (at 2 years), then 77 and 70 %, 6 and 12 years postoperatively. Mean BMI (kg/m^2^) decreased from 46 to 26.6 and was 28.5 and 29.9 at those time frames. Remission or improvement of comorbidities was achieved in most patients. The quality of life index was satisfactory in all parameters from 6 months onwards.

**Conclusions:**

Laparoscopic OAGB is safe and effective. It reduces difficulty, operating time, and early and late complications of Roux-en-Y gastric bypass. Long-term weight loss, resolution of comorbidities, and degree of satisfaction are similar to results obtained with more aggressive and complex techniques. It is currently a robust and powerful alternative in bariatric surgery.

## Introduction

Obesity has become one of the fastest-growing and greatest health problems in both developed and developing countries [1]. Morbid obesity (MO) leads to complications affecting nearly every organ system and a decrease in life expectancy as well [2]. Operative treatment is the most effective therapy available for MO. It enhances durable excess weight loss (EWL), eliminates (or ameliorates) comorbidities, improves quality of life (QoL), and lengthens life span [3]. The increasing demand for bariatric surgery (BS) has encouraged many digestive surgeons and laparoscopic experts to enter the field. Today, alternatives range from “simple” restrictive models to “complex” operations that radically alter gastrointestinal (GI) structure and function.

Laparoscopic mini-gastric bypass (MGB) was proposed as a simple and effective treatment for MO [4]. After two decades performing both open and laparoscopic BS, we adopted the MGB concept but developed adjustments to counteract its major criticism (namely alkaline reflux and its consequences). In our original publication [5], the term “one-anastomosis gastric bypass (OAGB)” was coined for this modified procedure. This study aimed to evaluate experience and long-term follow-up (FU) in a large cohort of patients with MO in whom laparoscopic OAGB was performed at a single institution.

## Patients and Methods

This is a retrospective review of a prospectively maintained database of 1200 consecutive patients with MO submitted to laparoscopic OAGB from July 2002 to October 2008. This comprises the initial part of our series, and data was analyzed after all patients completed a FU of 6 to 12 years. Patient inclusion was according to criteria by the National Institutes of Health Development Panel (body mass index (BMI) >40 kg/m^2^ or BMI >35 kg/m^2^ with severe related comorbidity) [6]. In agreement with current recommendations, patients with class I obesity and metabolic comorbidities were also included [7]. Ideal body weight was determined according to Metropolitan Life Insurance Company 1983 height/weight tables [8]. Excess weight (EW) was defined as the difference between a patient’s weight and the theoretical medium-frame ideal body weight. The percent of EW lost (%EWL), change in mean BMI, and mean percent of excess BMI lost (%EBMIL: initial BMI − current BMI / (initial BMI − 25) * 100) were used to evaluate weight loss (WL) [9]. Reinhold’s classification was used to define the effect of BS. Good and excellent results (EWL > 50 %) were defined as successful treatment [10]. Remission of comorbidities was considered when no medications and/or support were needed, and improvement when these latter decreased in number and/or dosing; this was corroborated clinically and/or biochemically. Written informed consent was obtained from all patients. The study was conducted after approval from the ethics committee and Institutional Review Board.

### Preoperative Assessment and Work-Up

Candidates were evaluated by a multidisciplinary medical unit and underwent preoperative psychological, nutritional, and comprehensive medical evaluations. A thorough assessment was performed of their general condition, associated illnesses, risk factors, mental status, motivations for BS, and ability to adhere to a postoperative regimen. Biochemical and radiological studies (abdominal ultrasound, chest x-ray, upper GI series), as well as endocrine and cardiopulmonary assessment, were performed. Supplementary tests related to existing comorbidities were carried out accordingly. Patients underwent respiratory physiotherapy and were encouraged to do physical exercise. In order to lose at least 10 % (20 % for BMI >50 kg/m^2^) of their EW preoperatively, they were submitted to 12 days of an 800-cal/day pure high-protein diet with micronutrients and vitamins (Vegefast-Complet®-Vegenat, Spain) followed by a complete liquid diet 5 to 7 days prior to operation. This was part of a prospective randomized controlled trial (RCT) comparing Vegefast-Complet*®* to a natural high-protein diet performed at our center [11]. Antithrombotic and antibiotic prophylaxis were given to all patients.

### Operative Technique

**Fig. 1:**
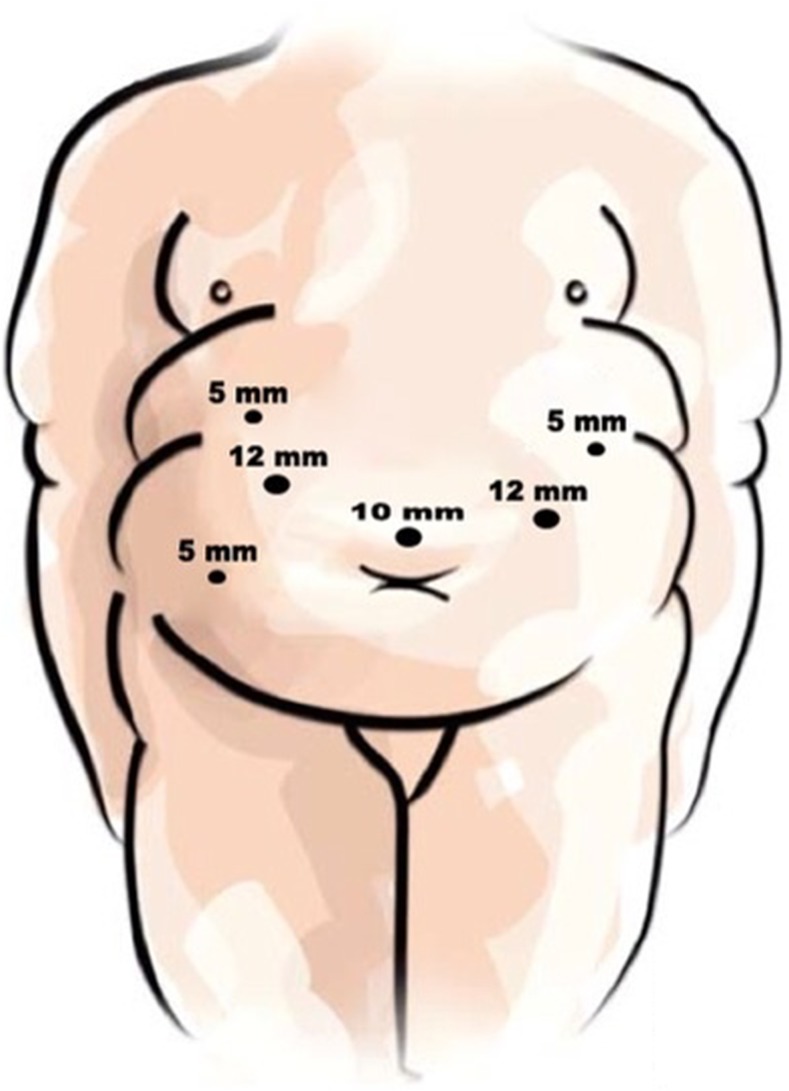
Sites for trocar placement in laparoscopic one-anastomosis gastric bypass which include one 10 mm (camera), two 12 mm (surgeon’s operating ports), and three 5 mm trocars for liver retraction and small bowel and stomach mobilization

**Fig. 2:**
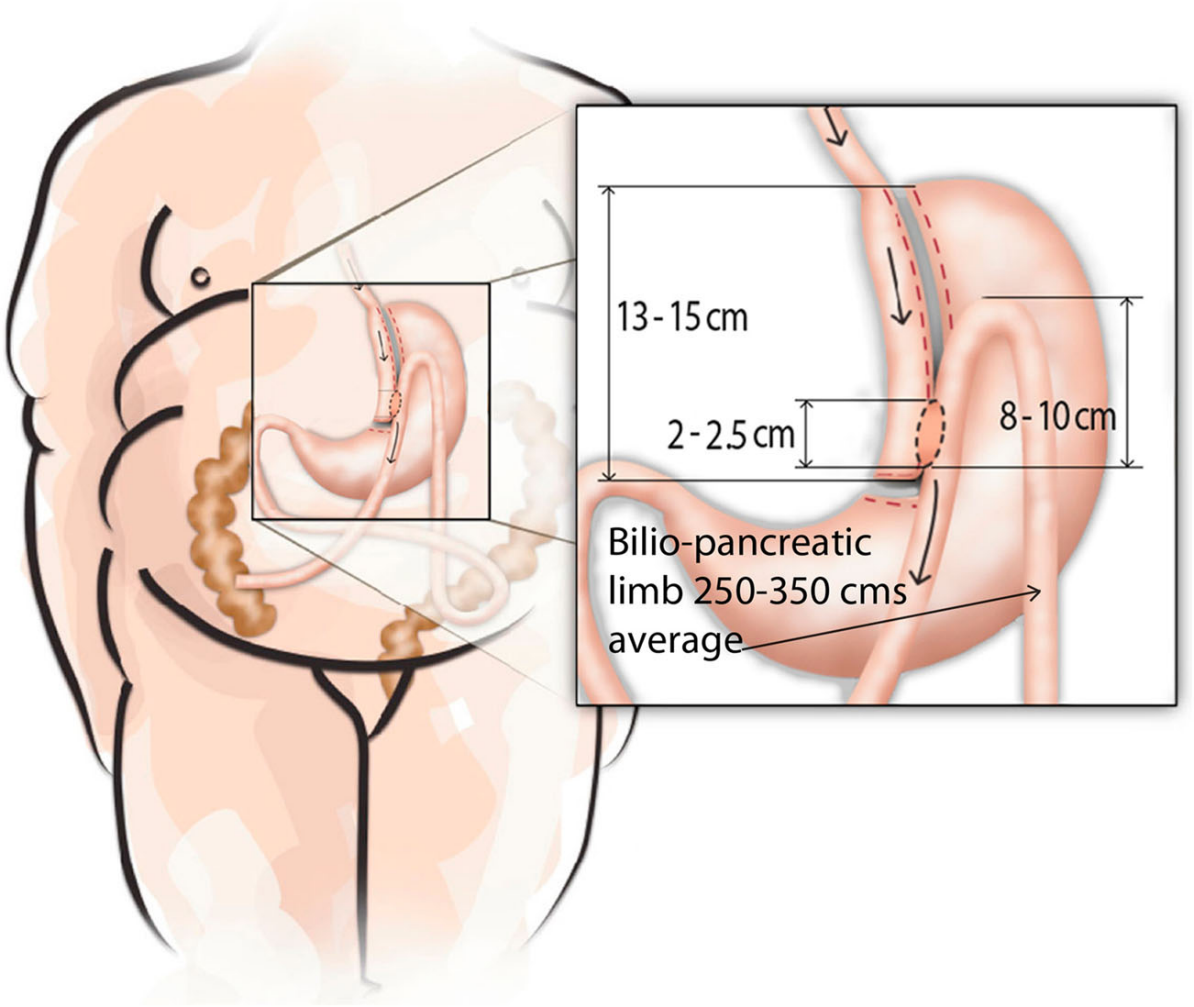
Diagrammatic representation of the one-anastomosis gastric bypass with gastric pouch (∼15 cm) and latero-lateral anastomosis. The afferent loop is suspended 8–10 cm above the anastomosis. The biliopancreatic limb averages 250–350 cm

All procedures were performed by a single surgeon (MC) and essentially the same surgical team. The patient is placed in a modified lithotomy position with the surgeon standing between the patient’s legs, the camera operator on the right side, and an assistant on the left. Six trocars are normally used (Fig. 1). OAGB is diagrammatically shown in Fig. 2. The first step consists of locating the Treitz ligament and measuring the jejunal loop to be bypassed which initially was ∼200 cm. After publishing results of our first 209 patients [5], we started to measure the whole small bowel (SB) routinely (Treitz to ileocecal valve), to determine the extent of bypassed SB (afferent limb) and common channel (efferent limb); we select the midportion and thus their lengths are usually similar (from ∼250 to 350 cm). For increasing BMIs, we add 10–50 cm of bypassed SB (with no specific formula) but always maintain at least ∼250–300 cm of common channel. Therefore, the extent of bypassed SB was based on total SB length and BMI for the last ∼1000 patients in this series. The patient is then placed in a 30° anti-Trendelenburg position. The angle of His is identified and the fat pad at the esophago-gastric (EG) junction explicitly dissected in order to visualize the diaphragm’s left crus for optimal endostapler positioning at this critical location. With associated hiatal hernia (very common in MO), this step includes section of peri-esophageal adhesions and phreno-esophageal ligament to reduce the hernia. Hiatal closure is performed selectively (marked enlargement). An automated camera-holding system (Lap Man®-Medsys, Belgium) is then installed and operated through a laser remote control (Lapstick®-Medsys, Belgium). Ultrasound shears (Autosonix®-Covidien, USA) are used to section the lesser gastric curvature’s fat and blood vessels at the level of the crow’s foot to enter the lesser sac. An endoscopic stapler loaded with a 45-mm/3.5-mm cartridge (Endo-GIA®-Covidien, USA) is inserted through the created opening and applied, sectioning the stomach horizontally. A 36-Fr double-lumen orogastric tube (Ref 340.36®, Vygon, France) is inserted to calibrate the gastric reservoir. Fatty tissue and fibrous adhesions between the posterior gastric wall and pancreas are dissected. Then, an endoscopic stapler loaded with 60-mm/3.5-mm cartridges (Endo-GIA®-Covidien, USA) is consecutively applied (usually three times), sectioning the stomach vertically and completing the gastric reservoir. The latter should be long, narrow, well vascularized, and easy to move caudally. The orogastric tube is removed and the previously chosen SB mobilized upward placing it without tension in an antecolic, antegastric position. Bivalving of omentum is especially required in superobese patients and those with central obesity. A continuous reabsorbable no. 2-0 suture (Polisorb®-Covidien, USA) is sewn in a latero-lateral position, securing the SB loop to the gastric reservoir’s staple line along ∼8–10 cm. Enterotomy and gastrotomy (distal reservoir) are done with ultrasound shears. An endoscopic stapler loaded with a 30-mm/3.5-mm cartridge (Endo-GIA®-Covidien, USA) is partially inserted (∼75 %) and applied between both, thus creating a gastroenteric anastomosis ∼2–2.5 cm long. Incisions on the anterior anastomotic wall are sutured with reabsorbable no. 2-0 (Polisorb®-Covidien, USA) interrupted stitches. These are also used to fix the (afferent) biliopancreatic (BP) SB loop in an upward direction (∼8–10 cm) to the excluded stomach, and also the common (efferent) SB loop to the excluded gastric antrum. These measures unload anastomotic tension, improve its orientation, and reinforce the antireflux mechanism. Anastomosis integrity is verified with a pneumatic test. Fibrin glue (Tissucol®-Baxter, USA) or surgical glue (Glubran 2 ®-Gem, Italy) is applied to the anastomotic surface and stapled regions of the gastric reservoir and remnant, and the greater omentum is tucked and adhered to them. Lastly, a penrose drain is positioned under the left hepatic lobe and brought out through the 5-mm right subcostal incision.

### Postoperative Management

An upper GI hydrosoluble contrast swallow (Gastrografin®-Bracco Diagnostics, Canada) was routinely performed the morning after to verify patency, rule out leaks, and provide a baseline postoperative “map.” The urine catheter was removed and the patient actively mobilized. A clear liquid diet was then started with swallows of ∼30 ml. The patients usually tolerated this regimen well, their drain was removed, and they were discharged ∼24 h postoperatively with specific indications regarding diet, activities, and medications. The drain was left in place and removed at the first office visit only in patients with unusual operative bleeding, a higher risk for postoperative bleeding (i.e., liver failure), and complex operative cases (i.e., reoperations and revisional BS).

### Medium- and Long-Term Follow-Up

Postoperative FU office visits for clinical and complete biochemical evaluation including macro- and micronutrients were scheduled 3, 6, 12, 18, and 24 months after BS, and yearly thereafter. In between these time frames, we kept close contact through telephone or e-mail. Due to our described referral pattern, we strove to keep an updated web page which included a blog and forum where patients could retrieve all kinds of information and maintain communication with any member of our team and with other patients as well. In regard to our electronic reports for our “remote” FU, concerning measurements and WL, we have tried to be as objective as possible and thus these include front and side full-length photographs with waist and hip measurements, and weight (either from an electronic scale report with name or a patient’s photo on a bathroom scale); moreover, the electronic FU report contains all information which would be evaluated during an in-office consultation including GI status, side effects and development of complications, eating and exercise habits, use of medications and supplements, and complete biochemical reports.

The patients followed a nutritional protocol designed at our center. Intestinal adaptation was usually completed within the first 3 months; however, the process could take up to 6 months or more, especially in cases of frequent dietary transgressions. From the 6th month onwards, the patients usually tolerated all kinds of food. They were administered proton pump inhibitors (PPIs) and sucralfate daily (first month), high-protein powder and calcium (first 3 months), and a daily mineral-vitamin supplement (first year), with maintenance doses recommended for life. Specific mineral and/or nutritional supplements were prescribed as needed for documented deficiencies (some starting even in the preoperative period).

### Gastrointestinal and Quality of Life Evaluation

Postoperative endoscopic (control) studies were planned for all patients completing a 5-year FU as well as for those referring persistent upper GI symptoms. Along with preoperative psychological evaluations, a QoL baseline assessment was applied using the Impact of Weight on QoL (IWQoL) survey [12]; this latter was repeated at periodic postoperative office visits and/or included in the electronic “remote” FU report.

## Results

### Demographics and Operative Data

There were 744 female (62 %) and 456 male (38 %) patients with a mean age of 43 years (range, 12–74). Mean preoperative BMI was 46 kg/m^2^ (range, 33–86) and mean preoperative EW was 65 kg (range, 34–220). Our cohort was composed of different subgroups which included 697 patients (58 %) with no previous or simultaneous abdominal operations (subgroup 1). Another 273 (23 %) had had prior open abdominal operations and thus required adhesiolysis of variable complexity, and a total of 203 (17 %) had abdominal operations performed simultaneously, particularly gallbladder removal and/or hiatal or ventral hernia repairs (subgroup 2). Finally, in 27 (2 %), laparoscopic OAGB was performed as a revision of other (failed) bariatric procedures (subgroup 3) including previous laparoscopic gastric bands (*n* = 13), as well as open vertical banded gastroplasties (*n* = 14). Perioperative results are shown in Table 1. Operative time (OT) varied among different subgroups. Mean length of stay (LOS) was significantly lower for patients without complications. Other results demonstrating quick patient recovery are outlined, as well as a summary of overall morbidity and mortality (further specified in Table 2). Noteworthy is the fact that we did not reoperate or have a record of any reoperation elsewhere for failure of the procedure.

**Table 1:** Overall perioperative characteristics of 1200 morbidly obese patients submitted to laparoscopic one-anastomosis gastric bypass (OAGB)

Variable	OAGB (*n* = 1200)
Mean (range) operative time (min)	
Subgroup 1	86 (45–180)
Subgroup 2	112 (95–230)
Subgroup 3	180 (130–240)
Mean (range) length of stay	
Uncomplicated patients (97.4 %)	24 h (15–120)
Complicated patients (2.6 %)	9 days (5–32)
Postoperative course	
Flatus passage	24–48 h
Analgesic use	24–36 h
CPAP/BiPAP needs^a^	24–72 h
Need for PPIs	1–3 months
Overall morbidity and mortality	
Major early morbidity	32 (2.7 %)
Major late morbidity	12 (1 %)
30-day readmission rate	10 (0.8 %)
Late readmission rate	13 (1 %)
30-day mortality	2 (0.16 %)
Reoperations for failure	0 (0 %)

*CPAP* continuous positive airway pressure, *BiPAP* bilevel positive airway pressure, *PPIs* proton pump inhibitors

^a^Only for preoperative CPAP/BiPAP users

**Table 2:** Complications and side effects following laparoscopic one-anastomosis gastric bypass (OAGB) in 1200 morbidly obese patients

	*n* (%)	Treatment/comments
Intraoperative complications requiring conversion to open surgery	4 (0.3)	
Intra-abdominal bleeding	2 (0.16)	LSML
EG junction perforation (calibration tube)	1 (0.08)	LSML (conversion to distal RYGB)
Incorrect gastric transection	1 (0.08)	LSML (conversion to distal RYGB)
Immediate postoperative complications resolved by open reoperations	6 (0.5)	
Intra-abdominal bleeding	2 (0.16)	LSML
Leaks (anastomotic/gastric reservoir)	2 (0.16)	LSML/one with prosthesis (placed radiologically)
Small bowel obstruction	1 (0.08)	LSML/afferent limb torsion
Partial necrosis of excluded stomach	1 (0.08)	LSML/patient died with nosocomial pneumonia
Immediate postoperative complications resolved by re-laparoscopy	10 (0.8)	
Intra-abdominal bleeding	7 (0.58)	Solved laparoscopically
Leak (anastomotic/gastric reservoir)	1 (0.08)	Solved laparoscopically/prosthesis (placed endoscopically)
Small bowel obstruction	1 (0.08)	Solved laparoscopically/adhesion (trocar incision-efferent limb)
Acute dilation excluded stomach	1 (0.08)	Solved laparoscopically
Early postoperative complications resolved conservatively	12 (1)	
Leaks (anastomotic/gastric reservoir)	10 (0.83)	Medical treatment (NPO and TPN)/endoscopic prostheses placed in two
Acute (postoperative) pancreatitis	1 (0.08)	Submitted to laparoscopic OAGB and cholecystectomy
Infected hematoma	1 (0.08)	Percutaneous drainage
Major late complications	12 (1)	
Gastroenteric (stomal) stenosis	6 (0.5)	Pneumatic endoscopic dilation (5), endoscopic coated prosthesis (1)
Anastomotic or marginal ulcer	6 (0.5)	Medical treatment/acute UGI bleed (5), chronic persistent pain (1); all had risk factors
Other complications and side effects		
Espohageal clinical reflux	26 (2)	Medical treatment
Malnutrition (protein)	14 (1.1)	Medical treatment/two readmitted for IV supplementation
Severe iron deficiency anemia	15 (1.25)	Medical treatment (parenteral iron)
Mild iron deficiency anemia	Up to 30 %	Medical treatment (oral iron)
Nausea/vomiting	6 (0.5)	Early readmission and medical treatment
Hair loss/iron/folate/B_12_ deficiencies	Variable	Medical treatment/improvement after intestinal adaptation
Diarrhea/bad fecal odor	Variable	Medical treatment/improvement after intestinal adaptation

*LSML* left subcostal mini-laparotomy, *RYGB* Roux-en-Y gastric bypass, *EG* esophago-gastric, *NPO* nil per os, *TPN* total parenteral nutrition, *UGI* upper gastrointestinal, *IV* intravenous

### Morbidity and Mortality

Complications and side effects are depicted in Table 2. Conversions to an open approach due to intraoperative problems and open reoperations due to immediate early complications were all performed through a left subcostal mini-laparotomy (LSML); this has been described elsewhere [13], and we utilized it routinely in the open bariatric era [14]. Intraoperative complications requiring conversion to an open approach occurred in four patients (0.3 %). Intra-abdominal hemorrhage was not adequately controlled by laparoscopic means in two; bleeding was from a gastric reservoir’s artery in one and from a ruptured vein in the posterior gastric wall in the other. The remaining two cases included one EG junction perforation by the calibration tube and one incorrect gastric transection in a patient with severe cardio-esophageal inflammation; both were related to technical difficulty during our learning curve and required conversion to distal Roux-en-Y gastric bypass (RYGB) with esophago-ileal anastomosis.

Early major complications requiring reoperations occurred in 16 patients (1.3 %) and included intra-abdominal bleeding (9), leaks (3), and early SB obstruction (SBO) (2). Rare complications included necrosis of excluded anterior gastric wall in one patient and acute dilation of excluded stomach in another. The former was in a patient with a BMI >70 who required a tracheostomy for intubation; the tube was displaced and severe abdominal pain developed few hours after the operation. Re-laparoscopy disclosed extensive necrosis of excluded stomach which was resected through LSML. In the other patient, excluded stomach and afferent limb were completely filled with BP secretion. Re-laparoscopy ruled out SBO and verified anastomosis integrity. Decompression was obtained through a nasogastric tube positioned into the afferent loop (removed upon the patient’s discharge) and a gastrostomy tube (removed on an ambulatory basis). Successful conservative treatment of early major complications was achieved in 12 patients (1 %), including leaks (10), acute pancreatitis (1), and infected hematoma (1).

Late complications included 6 stomal stenosis (0.5 %), 4 successfully treated through endoscopic dilation (single session) ∼2 to 3 months after operation. Another patient (lost to FU) was submitted at another hospital to repeated dilations and suffered a perforation that required urgent operative treatment. A further patient required a temporary prosthesis due to failed endoscopic treatments performed elsewhere 2 years after the initial operation. A total of 6 patients (0.5 %) developed anastomotic or marginal ulcers (MU); 5 were acute and presented without warning signs or symptoms with upper GI bleeding. Another presented more chronically with persistent epigastric pain. All had risk factors such as *Helicobacter pylori* (HP), and also (despite written and verbal warnings) chronic ingestion of aggressive medications, as well as alcohol (distilled) and tobacco consumption; they were managed conservatively.

Sporadic clinical reflux was reported by 26 patients (2 %). The few episodes were associated with dietary transgressions (always at night). Endoscopic studies revealed the presence of some bile in the stomach with mild to moderate pouch gastritis, but did not document any esophagitis. Treatment included dietary and healthy life recommendations, continued FU by our nutritionists, PPIs (40 mg/day for 6 months), and sucralfate (1 g before every meal and before bedtime for 3 months, followed by 1 g before bedtime for another 3 months). An upper GI endoscopy was ordered afterwards; all patients healed or significantly improved. Depending on their course, FU endoscopic studies were performed annually in some patients. Moreover, at completion of a 5-year FU, we planned screening endoscopic studies for all patients and were able to obtain them in 265 (22 %). Most other patients were completely asymptomatic and did not want the study. There were no significant findings; importantly, there were no cases of esophageal reflux and/or esophagitis or signs of acute or chronic stomal or MU. Mild to moderate pouch gastritis was found in 21 (8 %) and presence of HP in 9 (3.4 %). These patients were treated as those with clinical reflux (see above) and HP eradication when needed.

Preoperative nutritional deficits were found in some patients including iron (∼10 %), vitamin D (∼15 %), and calcium (∼4 %). After OAGB, a few patients developed excessive WL and/or nutrient deficits (usually within the first 2–3 postoperative years). Table 3 depicts the percentage of patients with specific deficiencies (values below normal range) at different points in time during our FU. Most were controlled and treated on an ambulatory basis and recovered with dietary recommendations and once intestinal adaptation was complete. However, a total of 14 patients (1.2 %) required further treatment for hypoalbuminemia; all received high-protein enteral supplements and pancreatic enzymes (Kreon*®-*Abbott, Germany) 10,000–25,000 IU with each meal during at least 3 to 6 months; 2 were readmitted and managed with IV albumin. Iron deficiency was rather common, especially in fertile women with copious menstrual bleeding. Up to one third required oral supplements beyond the expected time for intestinal adaptation, and 15 patients (1.3 %) required parenteral iron. Among liposoluble vitamins, vitamin D insufficiency was present in more than half of our patients at 3 years and one third in the long term; this required continuous supplementation in ∼20 % of them. Longer supplementation was also needed for vitamins A and K in ∼3 and 0.5 %, respectively. Deficits in hydrosoluble vitamins were basically found in B_9_ (folic acid) and B_12_; supplements were needed in ∼15 % at 3 years and 2 % in the longer term. An initial higher rate found in B_9_ (Table 3) was probably a reflection of preoperative deficiency. Calcium deficit was found in ∼8 % during the first 2 years and decreased thereafter, but persisted in ∼2 % in the longer term; supplements were especially recommended to post-menopausal women. Zinc and copper were needed in ∼5 and 3 % in the long term only in women. Specific long-term phosphorus, magnesium, and manganese supplementation has not been needed.

**Table 3:** Percentage of patients with nutritional deficits (lab values below normal) at different point intervals after one-anastomosis gastric bypass (OAGB)

Interval after OAGB (months)	3	6	12	18	24	36	60	120
Vitamin/nutrient								
A	0	0	0.9	1.8	1.6	2.1	0.8	0
D	18	21.4	32.3	19	38.3	54.9	45.7	32
E	0	0.2	0.1	0.1	0.2	0.1	0	0
K	0	0.1	0	0	0.3	0.5	0.1	0
B_1_	0	0.1	0.2	0	0	0	0	0
B_6_	0	0	0.2	0.2	0	0	0	0
B_9_ (folic acid)	32.5	17.6	18.6	24.1	22.2	18.2	9.8	2.3
B_12_	4.7	4	10.3	7.9	21.9	17.1	16.3	2.5
Iron	11.5	15.5	13.5	18.6	20.9	24.9	34.2	12.7
Calcium	4.3	8.1	6.7	5.6	2.9	1.3	2.1	1.9
Albumin	0.3	0.8	1.2	1	0.7	0.5	0.3	0

As in other malabsorptive procedures, soft stools, increased bowel gas, and a bad fecal odor were present in most patients, especially those consuming fatty and pure carbohydrate foods. Bismuth salts, activated carbon, and simeticone usually controlled symptoms well, and they usually improved progressively with intestinal adaptation. These nutritional complications and side effects were not significantly different when comparing our first 209 patients [5] to the more malabsorptive group of patients operated on thereafter (see “Operative Technique”).

The 30-day readmission rate was 0.8 % (10 patients). Two had SBO and were reoperated on. All others were successfully treated conservatively during 24–48 h; 6 had persistent nausea and vomiting, 1 had a single episode of hematemesis and had a stress ulcer at endoscopy, and 1 had an abstinence syndrome from psychiatric medication with extreme anxiety. Late readmissions were required in 13 patients (1 %) for stomal stenosis (6), GI bleeding due to MU (5), and malnourishment (2). Two patients died in this series (0.16 %); both had superobesity, multiple comorbidities, and risk factors. One suffered a pulmonary thromboembolism 3 days after BS (without warning symptoms or additional postoperative complications). The other suffered gastric wall necrosis, was reoperated on, and developed refractory nosocomial pneumonia. Both deaths occurred during the initial part of our series [9], and there were no other casualties in >1000 patients operated on thereafter.

### Follow-Up, Weight Loss, and Quality of Life

Table 4 depicts the number of patients operated on per year; a progressive increase was observed from 2002 to 2008. Also shown is accrual of patients for FU either as in-office visits or through electronic means. Although (as expected) there is a gradual decrease in number available for this long-term FU, at least 50 % of those operated on >12 years ago were being followed up, and we had information from 7 out of 10 among the whole group of 1200 patients. Our “remote” electronic FU reports were as objective as possible (see “Patients and Methods”); overall, outcomes for this group were not significantly different from those found in patients seen directly at our office. During this FU period, 15 (1.25 %) patients died from unrelated causes; these included aviation and auto accidents (4), lung cancer (2), surgical complications from unrelated operations several years after OAGB (2), family abandonment and suicide (2), myocardial infarction 5 years after OAGB (1), complicated appendicitis (1), complicated Guillain-Barre syndrome (1), unspecified autoimmune disease (1), and prostate cancer (1).

**Table 4:** Follow-up (FU) of number and percentage of patients at each year from July 2002 to October 2008

Years of FU (year of operation)	Operations/year	Physical (on-site) FU/year	Electronic FU/year	Total FU/year	No. of operations^a^	No. of patients in FU^a^	FU (%)^a^
6 years (2008)	268	148 (55 %)	85 (32 %)	233 (87 %)	268	233	87
7 years (2007)	302	112 (37 %)	102 (34 %)	214 (71 %)	570	447	84
8 years (2006)	248	79 (32 %)	81 (33 %)	160 (65 %)	818	607	74
9 years (2005)	146	44 (30 %)	53 (36 %)	97 (66 %)	964	704	73
10 years (2004)	92	23 (25 %)	32 (35 %)	55 (60 %)	1056	759	72
11 years (2003)	86	17 (20 %)	34 (40 %)	51 (59 %)	1142	810	71
12 years (2002)	58	12 (21 %)	17 (29 %)	29 (50 %)	1200	839	70
Total	1200	435 (36 %)	404 (34 %)	839 (70 %)			

^a^Cumulative

Depending on initial (preoperative) EW, patients lost a mean of ∼15–20 kg in the first month and ∼30–40 kg in the first trimester. Table 5 outlines evolution of WL expressed in various forms at different point intervals. The number and percentage of patients followed up at each time interval are included; only from 13 % (at 6 years) to 30 % (at 12 years) of our cumulative number of patients were lost for FU. Substantial WL was documented for most patients; through time, there was a slight weight increase in a few, which was not clinically relevant. Thus, EWL was maintained in most of our patients and according to Reinhold’s classification our results ranged from good (EWL >50 %) to excellent (EWL >75 %), and a long-term successful treatment (EWL >50 %) was achieved in almost all patients.

**Table 5:** Weight loss evolution after one-anastomosis gastric bypass (OAGB) in 1200 morbidly obese patients

Years of FU (year of operation)	Cumulative FU No. of patients (%)	Weight (kg)^a^	BMI (kg/m^2^)^a^	%EBMIL^a^	%EWL^a^
Preoperative		124 (82–308)	46 (33–86)		
6 years (2008)	233 (87)	68	28.54	83.09	77
7 years (2007)	447 (84)	69	28.74	82.89	76
8 years (2006)	607 (74)	71	29.32	79.38	73
9 years (2005)	704 (73)	72	29.64	77.85	72
10 years (2004)	759 (72)	73	29.89	76.60	70
11 years (2003)	810 (71)	73	29.89	76.60	70
12 years (2002)	839 (70)	73	29.95	76.30	70

*FU* follow-up, *BMI* body mass index, *%EBMIL* percentage excess BMI lost, *%EWL* percentage excess weight lost

^a^Mean

The effect of OAGB on comorbidities is shown in Table 6. The number of patients carrying each related disease preoperatively is included and varied widely. Figures in the columns “remission” and “improvement” represent what was recorded at the last patient evaluation (either as in-office or electronic FU). Severe metabolic comorbidities such as type II diabetes mellitus, insulin resistance, hypertension, and sleep apnea either totally resolved or substantially improved (most from the first day after BS). Remission was also demonstrated in most patients for other metabolic conditions like hyperlipidemia and liver steatosis when the first biochemical tests were ordered at the 3rd postoperative month. Benefits were also evident for patients with “mechanical” complications related to MO (osteoarthritis, urinary incontinence, and respiratory insufficiency). Noteworthy is the fact that 53 % of our patients had gastroesophageal reflux disease (GERD) of some degree and all were relieved after the operation. Significant improvement in QoL and in all but one IWQoL survey parameter was found 3 months after BS. The item “comfort with food” also improved markedly but only 6 months after the operation; moreover, problems with it were referred to as “insignificant” at the 1-year office visit, when no patients reported food intolerance of any kind. This improvement in QoL remained through time.

**Table 6:** Outcomes of one-anastomosis gastric bypass (OAGB) on comorbid conditions in 1200 morbidly obese patients

Comorbidity	No. with comorbidities (%)	Remission (%)	Improvement (%)
Type II diabetes mellitus	180 (15)	94	6
Fasting glucose impairment	216 (18)	100	–
Hypertension	387 (32)	94	6
Hyperlipidemia	673 (56)	96	4
Gastroesophageal reflux disease	636 (53)	92	8
Sleep apnea	1113 (93)	90	10
Osteoarthritis	1016 (85)	18	82
Urinary incontinence	60 (5)	22	78
Shortness of breath on exertion	1016 (85)	100	–
Fatty liver	1200 (100)	100	–
Polycystic ovarian disease	180 (15)	–	100

## Discussion

Widespread interest of specialists and demand from referring physicians and patients have created a soaring increase in the practice of BS. There are pros and cons in every operation; in general, safety is inversely related to effectiveness. While purely restrictive procedures have not proven to be effective especially in achieving substantial long-term EWL and metabolic benefits [15, 16], more complex malabsorptive operations have shown excellent EWL and remission of comorbidities [16], but at the cost of long learning curves, more complications, and less reproducibility [17]. As a mixed procedure, RYGB has been considered by many the “gold standard” [18] and was in fact our procedure of choice during the open era and beginning of the laparoscopic one. Laparoscopic RYGB, however, is a technically demanding procedure with a relatively high morbidity and need for reoperations (especially during its steep learning curve) [19]. Weight regain (WR) has also been an issue found in series with a long-term FU [20].

When MGB was first reported [4], we embraced the possibility of performing an operation with all benefits of a mixed procedure with arguably less complexity. As others, we were concerned about alkaline reflux and its potential consequences and thus developed modifications in order to provide an antireflux mechanism [5]. The scarcity of reports in MGB/OAGB has changed. There are now several publications of series [21–26], comparative studies [27–29], and RCT [30]; these types of studies have also been done with emphasis on the metabolic syndrome [31–40], and there are even systematic and integrative reviews [41–44]. However, few groups have studies of >1000 patients and most lack long-term FU; hence, we decided to analyze the first 1200 consecutive patients in our experience, who were operated on between 2002 and 2008, thus completing a 6- to 12-year FU.

### Perioperative Outcomes

Advantages ascribed to the procedure [4, 42–44] were evident from the perioperative period in our patients (Table 1). Recovery was usually quick and uneventful with rapid ambulation, flatus passage, and almost no need for analgesics; this led to a reduced LOS, which for our non-complicated cases (97 %) has been one of the shortest reported thus far [42, 44]. Although OAGB provides reduced difficulty compared to complex procedures, we believe it should not be promoted as a very simple, easy, and rapid operation. Our OT varied for each subgroup (Table 1), and was similar to other reports [42], but definitely fell short compared to the original MGB’s “thirty minute case” [4, 21]. Besides the fact that speediness is not among our goals, several modifications in OAGB prove time consuming.

### Safety

As others [21–26, 42–44], we found OAGB to be a rather safe operation. There were 32 (2.7 %) early and 12 (1 %) late major complications (Table 2). Almost 70 % of the former were treated conservatively or through re-laparoscopy, and all late complications were treated conservatively. Specific analysis led to interesting findings.

### Early Complications

#### Leaks

Leaks were the most common complication. However, our rate (1 %) is within range or lower than that of other MGB/OAGB [42–44], RYGB [45], and laparoscopic sleeve gastrectomy (LSG) series [46]. Experience was noted by a decrease from 1.9 % in our first 209 patients [5] to 0.9 % in those operated on thereafter. Most of them (77 %) were managed conservatively; this is not common in other procedures, where leaks may be quite difficult to treat and even fatal [16, 45]. Although some claim BP secretion in the anastomotic region may result in leaks after MGB/OAGB harder to manage [47], this did not happen. Since there is no enteric sectioning and all intestinal arcade supplies the area, blood flow in MGB/OAGB may hasten tissue healing; also, a much longer pouch and OAGB’s antireflux mechanism provide less mesenteric and vascular traction (Fig. 1). These anastomotic characteristics may have contributed to our favorable results with non-operative treatment. A less conservative approach was reported by Rutledge [21] who had a 1.1 % leak rate and all patients were reexplored through laparoscopy. Chevallier et al. [26] had 6 leaks (0.6 %); 3 were anastomotic, and all were reoperated on. In contrast, Musella et al. [24] had 10 leaks (1 %). They divided them according to origin; all 5 gastric pouch leaks needed operative treatment, but 2 gastric remnant and 2 of 3 anastomotic leaks were treated conservatively. The lowest leak rate from large MGB/OAGB series was reported by Kular et al. [25] with only 2 leaks in 1054 patients (0.1 %).

#### Bleeding

Intra-abdominal bleeding was the second most common complication; its incidence (0.9 %) compares favorably with other series [42, 44] and also decreased with time from 1.9 % [5] to 0.7 %. Intra-abdominal bleeding should be differentiated from intraluminal GI bleeding. Chances for conservative and/or endoscopic management in the former are usually higher [24, 25]. Those with hemoperitoneum may be managed non-operatively based on clinical grounds. Commonly, bleeding ceases spontaneously and staple lines are assumed to be the source [23].

#### Small Bowel Obstruction

In MGB/OAGB, there are no mesenteric defects to close, and as most surgeons accustom [26, 28, 41–44], we did not close Petersen’s defect. In spite of this, and as has been reported [21–30, 42–44], there were no internal hernias. The only 2 cases (0.16 %) of SBO were due to other causes. This represents a great advantage compared to laparoscopic RYGB, where internal hernias have been reported in up to 16 % of patients [20]; some present many years after with symptoms that range from simple chronic abdominal pain to frank bowel necrosis, leading to difficulties for a timely diagnosis and sometimes fatal outcomes [48].

### Late Complications

#### Stomal Stenosis

All 6 (0.5 %) gastroenteric (stomal) stenosis presented during the initial part of our series when anastomotic size ranged from 1.5 to 2 cm. After increasing to ∼2.5 cm, we had no further problems. All were successfully managed with pneumatic endoscopic dilations. Rate is within range of other MGB/OAGB series and much better than most RYGB series where strictures complicate up to 27 % of patients [49]. Although anastomotic tension, ischemia, and subclinical leaks may cause these lesions, another potential contribution is that standard RYGB includes a narrower (∼1.2 cm) anastomosis [49]. Moreover, although most can be treated with endoscopic dilations, up to a third may require a reoperation [50].

#### Marginal Ulcer

Critiques to MGB/OAGB emphasized it would lead to a higher rate of MU and with less responsiveness to medical management [47]. Recent systematic reviews report an incidence of 0.6–4 % for large series [42, 44] which is less than that found after RYGB which has a wide range between 0.6 and 25 % [51]. Various risk factors independent of bile reflux (BR) have been identified [51]. Increased acid production in an oversized pouch is a potential cause, and some hypothesized presence of bile within the anastomotic area in MGB/OAGB may actually have a protective effect buffering acid’s ulcerogenic action [26]. Our MU rate of 0.5 % is one of the lowest reported for any type of gastric bypass. We meticulously explain and stress avoidance of risk factors to our patients; whether our antireflux mechanism had further beneficial effects is difficult to ascertain. Moreover, this longer FU demonstrates they were as responsive to medical therapy as those arising after RYGB. Our patients and those in most MGB/OAGB series [26, 28, 42, 44] have responded to PPIs, sucralfate, and HP eradication.

#### Bile Reflux

The main condemning argument against MGB/OAGB has been the potential for BR and its consequences. This has been dealt with thoroughly previously [24–28, 41–44]; gastric and esophageal BR must be analyzed separately. Although BR to the stomach may be frequent both physiologically [52] and after some operations [53], symptomatic, endoscopic, and histologic repercussions have neither been relevant nor conclusively proven [54]. BR to the esophagus, on the other hand, may have all, at least experimentally [55]. Although it was common, and the main reason to abandon Mason’s loop [56], MGB’s anatomical configuration makes esophageal BR highly improbable. Our OAGB (antireflux technique) takes this a step further.

Symptomatic gastric and/or esophageal BR after MGB/OAGB has been rare [26, 28, 41–44]. BR was referred by 2 % of our patients. Symptoms were sporadic (2–3 times/year) and included a bitter and/or sour sensation, sometimes reaching the throat, usually at night after dietary transgressions. Medical treatment was successful in all; to our knowledge, no patient has been reoperated on elsewhere due to “intractable” BR. Further seeking BR, we studied our first 20 patients with endoscopy and 24-h pHmetry, 12 and 18 months after BS; results were all normal [5]. We extended this assessment and planned upper GI endoscopies for all those reaching a 5-year FU. Almost 80 % did not accept, mostly because they were completely asymptomatic. Findings included mild to moderate pouch gastritis (8 %) and HP (3 %), but there were no worrisome endoscopic or histological changes.

Postoperative ancillary studies after MGB/OAGB have been carried out by others. Chevallier et al. [57] found no esophageal changes or significant findings in asymptomatic patients. Musella et al. [24] performed endoscopies in 26 (3 %) symptomatic patients with prolonged dyspepsia, epigastric pain, heartburn, and vomiting and found anastomotic ulcers (1.7 %) and biliary gastritis (0.9 %), but no other significant finding. More recently, Tolone et al. [58] evaluated EG junction function pre- and postoperatively through endoscopy, high-resolution impedance manometry, and 24-h pH-impedance monitoring; results were compared with patients submitted to LSG. After MGB, there was no heartburn/regurgitation, esophagitis, biliary gastritis, or presence of bile; also, manometric patterns did not vary (from preoperative). Intragastric pressure, GE pressure gradient, and reflux events (acid, weakly acid, and even weakly alkaline) all significantly diminished. In contrast, due to the pylorus (functional obstruction), LSG led to a significant elevation in all these parameters, and in esophageal acid exposure. In fact, many consider LSG unsuitable for patients with GERD and/or Barrett’s esophagus [59]; reoperations for intractable GERD are not uncommon [60].

In contrast, reoperations after MGB/OAGB due to “intractable” BR are rare, especially when standard operative techniques are performed [26, 61, 62]; this has ranged from 0 % (most series) to 0.7 % [42, 44]. Critics have also published reoperations due to BR [47]; however, as they accept, the denominator in their series is not known. Moreover, in no case has it been described exactly what “intractability” meant. It is important to be familiar with postoperative phases of GI adaptation to MGB/OAGB, so as not to blame BR as the culprit of any dyspeptic symptom. Kular et al. [25] recently defined BR after MGB as bilious vomiting and/or documented bile in the esophagus on upper GI endoscopy with presence of GERD-like symptoms, and proposed differentiating it from vague symptoms that characterize “dyspepsia.” A clear definition must be made in regard to symptoms, diagnostic approach, and medical treatment before considering a reoperation due to “intractability.” When needed, these latter have included conversion to RYGB (with or without gastric pouch shortening) and Braun entero-enterostomy [26, 47, 61, 62]. Since these few reoperations are usually performed in patients who have already achieved significant EWL and metabolic benefits, they are oftentimes technically not demanding and very effective.

#### Bile Reflux and Gastroesophageal Cancer

The long-lasting association between gastric BR after gastrectomy and gastric cancer has not been shared by many [63]. Recent comprehensive reviews conclude BR is only one of many potential risk factors involved in gastric stump carcinoma [64]; these act as confounding variables which are difficult to isolate to reach valid conclusions. Perhaps HP is the most significant not considered in older studies [65]. Since GE cancer may appear 20 to 30 years postoperatively, the fact that it has not been found after MGB/OAGB [41–44] may seem not enough evidence. However, even the highly criticized and abandoned Mason’s loop with its proven BR has not been associated with esophageal cancer, and there is only one case of gastric pouch cancer 26 years after [66]. The other 3 reports of cancer after Mason’s loop were in the bypassed stomach (not related to BR), and they have also been found after other bariatric operations [67].

Cancer arising in the gastric pouch or esophagus has been reported after BS [67], but never after MGB/OAGB. The only case of cancer reported thus far also originated in the excluded stomach in an Asian patient 9 years after MGB [68]. In spite that in vitro bile in the esophagus can stimulate production of inflammatory mediators and lead to changes in genetic expression to intestinal metaplasia [55], clinical studies have not demonstrated an association between gastric bile and Barrett’s esophagus [69]. Moreover, bile in the distal esophagus in patients with GERD seems not to correlate with Barrett’s esophagus as acid reflux does [70]. It is interesting to note that in spite that LSG has been conclusively associated with de novo or exacerbation of GERD [15], and GERD has been proven to lead to Barrett’s esophagus and cancer [71], MGB/OAGB has been the procedure under rigorous scrutiny for a potential cancer risk, which so far has not been demonstrated. Cancer in the distal esophagus has already been reported not only after LSG but also after other BS including RYGB [67].

#### Malabsortion/Malnutrition

Over time, we have progressively increased the extent of bypassed SB from ∼200 cm to a range of 2.5 to 3.5 mts, based both on total SB length and BMI (see “Operative Technique”). This “tailoring” has also been performed by other MGB/OAGB surgeons [22, 23, 34]. Although increased malabsorption could theoretically lead to more side effects and malnutrition, only 14 (1.1 %) patients suffered protein malnutrition. This rate is comparable to that reported in other MGB/OAGB [26, 28, 42, 44] and RYGB series [20]. Our strict postoperative regimen may have contributed to these figures, and not surprisingly all those with protein malnutrition admitted not following our program.

Malabsorption is only one of many factors that lead to malnutrition; among others, these include psychologic, personal, family, social, and even economic. Malnutrition can thus be seen after procedures which entail none or less malabsorptive components [20, 72, 73]. In our experience, malnutrition has been temporary; after our support program including IV therapy followed by a strict program of enteral supplementation and counseling (aimed at improving all other factors that influence nutritional status), and once intestinal adaptation is reached [74], it has posed no further problems. Long-term albumin therapy has not been required, and so far, we have not documented any case needing reversal or conversion to other operations for this reason in our group. Chevallier et al. [26] have also not reversed any patient but report malnutrition in 2 (0.2 %) who were under evaluation for a reoperation. They also noted deficiency problems always involved noncompliant patients. In the very few cases of reoperations reported by others, alternatives have included reversal to a normal anatomy or conversion to LSG. In his larger study, Rutledge [21] reported excessive WL in 1 % and selected reversal as their reoperation of choice. Lee et al. [61] revised 23 of 1322 patients (1.7 %); the most common cause was malnutrition in 9 (0.7 %). They recommended conversion to LSG due to efficacy in improving malnutrition without regaining body weight. Noun et al. [23] reported excessive WL in 4 (0.4 %) patients with reversal in 2 and conversion to LSG in the other 2. The Italian group [24] submitted 7 of 818 patients (0.8 %) to late reoperations; indication was EWL > 100 % in only one (0.1 %). All these groups agree a laparoscopic approach with its attendant benefits in this kind of reoperations was feasible and technically not demanding.

#### Other Complications and Side Effects

Iron deficiency anemia (IDA) has been rather common after any type of gastric bypass [72, 73]. Although we originally published rates of 1 % in severe IDA (requiring parenteral iron) and 7 % in moderate IDA (needing oral iron) [5], figures increased to 1.25 % and up to 30 %, respectively, after this longer FU. Hair loss and mild deficiencies in micronutrients were noted during the first months but usually subsided once intestinal adaptation was reached. Very few patients required supplementation beyond this time (especially of folate, calcium, and vitamin B_12_). Since almost half of our patients came from northern Spain where sunlight is limited, vitamin D has been a common deficit both pre- [75] and postoperatively. Supplementation is currently started prior to OAGB and is continued long term in up to 20 % of those patients.

In regard to other GI side effects, nausea and vomiting were rare, but we did have to readmit 6 (0.5 %) patients for medical treatment and reassurance in the early postoperative period. As in other MGB/OAGB series [26, 28, 42, 44], dumping was uncommon. Permanent contact of food with BP secretions leading to a slower absorption of sugars may be a plausible explanation which would need further studies for confirmation [73]. When present, symptoms were transient and well controlled with dietary adjustments. This contrasts with RYGB where dumping is quite common and even considered an asset in order to limit a patient’s consumption of sugars and carbohydrates, but may end up as a considerable problem affecting digestive comfort and QoL [76].

### Efficacy

#### Weight Loss

OAGB had a greater WL compared to any purely restrictive operation [3, 15, 16], standard RYGB [3, 16, 20], and even some series of MGB [42, 44]; WL curves were steeper, frequently reaching ideal weight, and similar results to those obtained with more complex malabsorptive techniques [3, 16]. Once WL stabilized, it was maintained through the years in most patients, with only subtle increases in some. Substantial WR requiring a reintervention was not evident in this series. Although inadequate EWL and/or considerable WR is especially seen in patients submitted to purely restrictive techniques [3, 15, 16], RYGB series with a long-term FU have reported them in ∼35 % and up to 20 %, respectively [20, 77]. This has led to modifications such as banded RYGB [78] and distal RYGB [79] in order to optimize EWL and its durability. Comparative studies have demonstrated superior efficacy of MGB/OAGB in terms of WL when compared to LSG [28], and either similar or greater WL when compared to RYGB [27, 29, 30].

#### Resolution of Metabolic and Other Comorbidities

Both MGB and OAGB comprise characteristics which are common in metabolic surgery. These include some form of restriction, and a long BP limb which bypasses the proximal gut and places food in the distal SB [80]. Its metabolic effect in our series was outstanding (Table 6) and similar to results obtained by others [31–40]. Metabolic comorbidities improved immediately and most subsided during the following weeks; this was corroborated clinically and biochemically during our FU. Once WL was being achieved, other “mechanical”-related comorbidities were also abated. We recently published results from selected patients included in the IFSO-European Accreditation Council (EAC) for Centers of Excellence database with insulin resistance/type 2 DM [32] or lipid profile derangements [36]. Substantial WL and significant improvement or full remission from their metabolic condition was observed in both series. Overall, comparative studies and RCT favor MGB/OAGB over purely restrictive operations and are similar to RYGB in their metabolic benefits [37–40].

### Follow-Up

Poor FU is definitely the “Achilles heel” in all BS. Since most patients came from different parts of our country, direct in-office consultation is difficult to accomplish. Nonetheless, we have been able to physically see >1/2 and >1/5 of our patients, 6 and 12 years after the operation, respectively (Table 4). Since our referral pattern was evident to us from the beginning, we strove to keep close contact with our patients through telephone or electronic means; also, “remote” reports have been as objective as possible and include all information that would be obtained directly at the office including photographs, questionnaires, and full biochemical tests. Certification by the IFSO-EAC as a Centre of Excellence and their rigorous and increasing requisites for recertification have validated our methods and continually support and guide our efforts to improve overall FU. The IFSO-EAC database indeed serves as the registry some colleagues have strongly recommended in order to have better insight in regard to this operation [47]. With all these resources, we have been able to gather information from ∼70 % of our patients through this long-term FU (Table 4).

### Name of the Procedure—a Final Controversy

A highly criticized concept, when initially proposed, is steadily being accepted worldwide. Centers of more than 25 countries are now performing it [81]. This has brought about a controversy regarding the name for the procedure, which has led to unfortunate confusions [82]. We recently presented our arguments on why we support views of others in considering OAGB as the only standing alternative name for MGB, in order to reconcile terms and facilitate issues in publishing future related courses and communications [83]. This was further supported at the 2015 IFSO meeting in Vienna where the academic MGB-OAGB International Club was founded [84]. We call again on the various bariatric teams that are performing the original MGB or our modified version, the OAGB, to aid in the dissemination and acceptance of this procedure by presenting and publishing their experiences and standardizing the name (to MGB/OAGB), in order for all of us to be recognized as a whole. Now that many of its controversies are being surpassed and the bariatric surgical community is accepting the procedure as a rational alternative in the bariatric repertoire, we should make all efforts in order to conciliate in regard to the name, avoid new disagreements, and work towards making MGB/OAGB mainstream in obesity and metabolic surgery.

## Conclusions

This group represents the original largest series, and with the longest FU of patients submitted to the so-called OAGB or BAGUA (*bypass gástrico de una anastomosis—*in Spanish), a modification of the MGB. It is also one of the largest series with longer FU among all centers performing the MGB/OAGB concept. OAGB provides reduced difficulty and operating time, and very low morbidity and mortality. Alkaline reflux (clinical and endoscopic) is very infrequent, mild, and treatable. Concerns regarding BR and its potential consequences currently seem unsubstantiated but await studies with even longer-term outcomes. So far, development of subsequent cancer has not been reported. Long-term substantial EWL, remission of comorbidities through its metabolic benefits, and degree of satisfaction are similar to the best results obtained with more aggressive and complex operations. OAGB is a safe and effective powerful alternative which is slowly (but surely) becoming mainstream in bariatric surgery.

## Abbreviations

OAGB: One-anastomosis gastric bypass
FU: Follow-up
BMI: Body mass index
MO: Morbid obesity
EWL: Excess weight loss
QoL: Quality of life
BS: Bariatric surgery
GI: Gastrointestinal
MGB: Mini-gastric bypass
EW: Excess weight
%EWL: Percentage of excess weight lost
%EBMIL: Percentage of body mass index lost
WL: Weight loss
RCT: Randomized controlled trial
SB: Small bowel
EG: Esophago-gastric
BP: Biliopancreatic
PPI: Proton pump inhibitor
IWQoL: Impact of weight on quality of life
OT: Operative time
LOS: Length of stay
LSML: Left subcostal mini-laparotomy
RYGB: Roux-en-Y gastric bypass
SBO: Small bowel obstruction
MU: Marginal ulcer
HP: *Helicobacter pylori*
GERD: Gastroesophageal reflux disease
WR: Weight regain
LSG: Laparoscopic sleeve gastrectomy
BR: Bile reflux
IDA: Iron deficiency anemia
EAC: European Accreditation Council

## References

[1] Malik VS, Willett WC, Hu FB (2013). Global obesity: trends, risk factors and policy implications. Nat Rev Endocrinol.

[2] Guh DP, Zhang W, Bansback N (2009). The incidence of comorbidities related to obesity and overweight: a systematic review and meta-analysis. BMC Public Health.

[3] Buchwald H, Avidor Y, Braunwald E (2004). Bariatric surgery: a systematic review and meta-analysis. JAMA.

[4] Rutledge R (2001). The mini-gastric bypass: experience with the first 1274 cases. Obes Surg.

[5] Carbajo M, Garcia-Caballero M, Toledano M (2005). One-anastomosis gastric bypass by laparoscopy: results of the first 209 patients. Obes Surg.

[6] Gastrointestinal surgery for severe obesity (1991). National Institutes of Health consensus development conference draft statement. Obes Surg.

[7] Buchwald H (2005). Consensus conference statement: bariatric surgery for morbid obesity: health implications for patients, health professionals, and third-party payers. J Am Coll Surg.

[8] Metropolitan Life Foundation (1983). Metropolitan height and weight tables. Metropolitan Life Foundation Statistical Bulletin.

[9] Deitel M, Gawdat K, Melissas J (2007). Reporting weight loss 2007. Obes Surg.

[10] Reinhold RB (1982). Critical analysis of long term weight loss following gastric bypass. Surg Gynecol Obstet.

[11] Carbajo MA, Castro MJ, Kleinfinger S (2010). Effects of a balanced energy and high protein formula diet (Vegestart complet®) vs. low-calorie regular diet in morbid obese patients prior to bariatric surgery (laparoscopic single anastomosis gastric bypass): a prospective, double-blind randomized study. Nutr Hosp.

[12] Kolotkin RL, Head S, Hamilton M (1995). Assessing impact of weight on quality of life. Obes Res.

[13] Jones KB (1998). The left subcostal incision revisited. Obes Surg.

[14] Carbajo MA, Martin del Olmo JC, Toledano M (2002). Left subcostal minilaparotomy in silastic ring vertical gastroplasty and transected Roux-en-Y gastric bypass. Obes Surg.

[15] Himpens J, Dobbeleir J, Peeters G (2010). Long-term results of laparoscopic sleeve gastrectomy for obesity. Ann Surg.

[16] Padwal R, Klarenbach S, Wiebe N (2011). Bariatric surgery: a systematic review and network meta-analysis of randomized trials. Obes Rev.

[17] Angrisani L, Santonicola A, Iovino P (2015). Bariatric surgery worldwide 2013. Obes Surg.

[18] Pope GD, Birkmeyer JD, Finlayson SRG (2002). National trends in utilization and in hospital outcome of bariatric surgery. J Gastrointest Surg.

[19] El-Kadre L, Tinoco AC, Tinoco RC (2013). Overcoming the learning curve of laparoscopic Roux-en-Y gastric bypass: a 12-year experience. Surg Obes Relat Dis.

[20] Higa K, Ho T, Tercero F (2011). Laparoscopic Roux- en-Y gastric bypass: 10-year follow-up. Surg Obes Relat Dis.

[21] Rutledge R, Walsh W (2005). Continued excellent results with the mini-gastric bypass: six-year study in 2,410 patients. Obes Surg.

[22] Lee WJ, Wang W, Lee YC (2008). Laparoscopic mini-gastric bypass: experience with tailored bypass limb according to body weight. Obes Surg.

[23] Noun R, Skaff J, Riachi E (2012). One thousand consecutive mini-gastric bypass: short and long-term outcome. Obes Surg.

[24] Musella M, Sousa A, Greco F (2014). The laparoscopic mini-gastric bypass: the Italian experience: outcomes from 974 consecutive cases in a multi-center review. Surg Endosc.

[25] Kular KS, Manchanda N, Rutledge R (2014). A 6-year experience with 1,054 mini-gastric bypasses—first study from Indian subcontinent. Obes Surg.

[26] Chevallier JM, Arman GA, Guenzi M (2015). One thousand single anastomosis (omega loop) gastric bypasses to treat morbid obesity in a 7-year period: outcomes show few complications and good efficacy. Obes Surg.

[27] Disse E, Pasquer A, Espalieu P (2014). Greater weight loss with the omega loop bypass compared to Roux-en-Y gastric bypass: a comparative study. Obes Surg.

[28] Jammu GS, Sharma R (2016). A 7-year clinical audit of 1107 cases comparing sleeve gastrectomy, Roux-en-Y gastric bypass, and mini-gastric bypass, to determine an effective and safe bariatric and metabolic procedure. Obes Surg.

[29] Lee WJ, Ser KH, Lee YC (2012). Laparoscopic Roux-en-Y vs. mini-gastric bypass for the treatment of morbid obesity: a 10-year experience. Obes Surg.

[30] Lee WJ, Yu PJ, Wang W (2005). Laparoscopic Roux-en-Y versus mini-gastric bypass for the treatment of morbid obesity: a prospective randomized controlled clinical trial. Ann Surg.

[31] Lee WJ, Wang W, Lee YC (2008). Effect of laparoscopic mini-gastric bypass for type 2 diabetes mellitus: comparison of BMI > 35 and <35 kg/m^2^. J Gastrointest Surg.

[32] Carbajo MA, Jimenez JM, Castro MJ (2014). Outcomes in weight loss, fasting blood glucose and glycosylated hemoglobin in a sample of 415 obese patients, included in the database of the European Accreditation Council for Excellence Centers for Bariatric Surgery with laparoscopic one anastomosis gastric bypass. Nutr Hosp.

[33] Coskun H, Hasbahceci M, Bozkurt S (2014). Effect of laparoscopic mini-gastric bypass on diabetes in morbidly obese patients. Eur J Laparosc Surg.

[34] García-Caballero M, Reyes-Ortiz A, Garcia M (2014). Changes of body composition in patients with BMI 23-50 after tailored one anastomosis gastric bypass (BAGUA): influence of diabetes and metabolic syndrome. Obes Surg.

[35] Guenzi M, Arman G, Rau C (2015). Remission of type 2 diabetes after omega loop gastric bypass for morbid obesity. Surg Endosc.

[36] Carbajo MA, Fong-Hirales A, Luque-de-Leon E (2016). Weight loss and improvement of lipid profiles in morbidly obese patients after laparoscopic one anastomosis gastric bypass: 2-year follow-up. Surg Endosc.

[37] Lee WJ, Lee YC, Ser KH (2008). Improvement of insulin resistance after obesity surgery: a comparison of gastric banding and bypass procedures. Obes Surg.

[38] Milone M, Di Minno MN, Leongito M (2013). Bariatric surgery and diabetes remission: sleeve gastrectomy or mini-gastric bypass?. World J Gastroenterol.

[39] Milone M, Lupoli R, Maletta P (2015). Lipid profile changes in patients undergoing bariatric surgery: a comparative study between sleeve gastrectomy and mini-gastric bypass. Int J Surg.

[40] Lee WJ, Chong K, Lin YH (2014). Laparoscopic sleeve gastrectomy versus single anastomosis (mini-) gastric bypass for the treatment of type 2 diabetes mellitus: 5-year results of a randomized trial and study of incretin effect. Obes Surg.

[41] Mahawar KK, Carr WR, Balupuri S (2014). Controversy surrounding ‘mini’ gastric bypass. Obes Surg.

[42] Mahawar KK, Jennings N, Brown J (2013). “Mini” gastric bypass: systematic review of a controversial procedure. Obes Surg.

[43] Lee WJ, Lin YH (2014). Single-anastomosis gastric bypass (SAGB): appraisal of clinical evidence. Obes Surg.

[44] Georgiadou D, Sergentanis TN, Nixon A (2014). Efficacy and safety of laparoscopic mini-gastric bypass. A systematic review. Surg Obes Relat Dis.

[45] Fernandez AZ, DeMaria EJ, Tichansky DS (2004). Experience with over 3000 open and laparoscopic bariatric procedures: multivariate analysis of factors related to leak and resultant mortality. Surg Endosc.

[46] Aurora AR, Khaitan L, Saber AA (2012). Sleeve gastrectomy and the risk of leak: a systematic analysis of 4,888 patients. Surg Endosc.

[47] Johnson WH, Fernandez AZ, Farrell TM (2007). Surgical revision of loop (“mini”) gastric bypass procedure: multicenter review of complications and conversions to Roux-en-Y gastric bypass. Surg Obes Relat Dis.

[48] Paroz A, Calmes JM, Giusti V (2006). Internal hernia after laparoscopic Roux-en-Y gastric bypass for morbid obesity: a continuous challenge in bariatric surgery. Ob Surg.

[49] Woodward GA, Morton JM, Deitel M, Gagner M, Dixon JB, Himpens J (2010). Stomal stenosis after gastric bypass. Handbook of obesity surgery.

[50] Sataloff DM, Lieber CP, Seinige UL (1990). Strictures following gastric stapling for morbid obesity: results of endoscopic dilatation. Am Surg.

[51] Coblijn UK, Goucham AB, Lagarde SM (2014). Development of ulcer disease after Roux-en-Y gastric bypass, incidence, risk factors and patient presentation: a systematic review. Obes Surg.

[52] Fuchs KH, Maroske J, Fein M (1999). Variability in the composition of physiologic duodenogastric reflux. J Gastrointest Surg.

[53] Atak I, Ozdil K, Yücel M (2012). The effect of laparoscopic cholecystectomy on the development of alkaline reflux gastritis and intestinal metaplasia. Hepato-Gastroenterology.

[54] Schindlbeck NE, Heinrich C, Stellaard F (1987). Healthy controls have as much bile reflux as gastric ulcer patients. Gut.

[55] McQuaid KR, Laine L, Fennerty MB (2011). Systematic review: the role of bile acids in the pathogenesis of gastro-oesophageal reflux disease and related neoplasia. Aliment Pharmacol Ther.

[56] Mason EE, Ito C (1967). Gastric bypass in obesity. Surg Clin North Am.

[57] Chevallier JM, Trelles N, Arienzo R (2011). Endoscopic findings after laparoscopic omega loop gastric bypass. Obes Surg.

[58] Tolone S, Cristiano S, Savarino E (2016). Effects of omega-loop bypass on esophagogastric junction function. Surg Obes Rel.

[59] DuPree CE, Blair K, Steele SR (2014). Laparoscopic sleeve gastrectomy in patients with preexisting gastroesophageal reflux disease a national analysis. JAMA Surg.

[60] Weiner RA, Theodoridou S, Weiner S (2011). Failure of laparoscopic sleeve gastrectomy—further procedure?. Obes Facts.

[61] Lee WJ, Lee YC, Ser KH (2011). Revisional surgery for laparoscopic minigastric bypass. Surg Obes Relat Dis.

[62] Luque-de-Leon E, Carbajo MA (2016). Conversion of one-anastomosis gastric bypass (OAGB) is rarely needed if standard operative techniques are performed. Obes Surg.

[63] Schafer LW, Larson DE, Melton LJ (1983). The risk of gastric carcinoma after surgical treatment for benign ulcer disease. A population-based study in Olmsted County, Minnesota. N Engl J Med.

[64] Sitarz R, Maciejewski R, Polkowski WP (2012). Gastroenterostoma after Billroth antrectomy as a premalignant condition. World J Gastroenterol.

[65] Seoane A, Bessa X, Alameda F (2005). Role of Helicobacter pylori in stomach cancer after partial gastrectomy for benign ulcer disease. Rev Esp Enferm Dig.

[66] Babor R, Booth M (2006). Adenocarcinoma in the gastric pouch 26 years after loop gastric bypass. Obes Surg.

[67] Scozzari G, Trapani R, Toppino M (2013). Esophagogastric cancer after bariatric surgery: systematic review of the literature. Surg Obes Relat Dis.

[68] Wu CC, Lee WJ, Ser KH (2013). Gastric cancer after mini-gastric bypass surgery: a case report. Asian J Endosc Surg.

[69] Taha AS, Angerson WJ, Morran CG (2003). Reflux and Barrett’s oesophagitis after gastric surgery—long-term follow-up and implications for the roles of gastric acid and bile in oesophagitis. Aliment Pharmacol Ther.

[70] Champion G, Richter JE, Vaezi MF (1994). Duodenogastroesophageal reflux: relationship to pH and importance in Barrett’s esophagus. Gastroenterology.

[71] Chang JT, Katzka DA (2004). Gastroesophageal reflux disease, Barrett’s esophagus, and esophageal adenocarcinoma. Arch Intern Med.

[72] Bernert CP, Ciangura C, Coupaye M (2007). Nutritional deficiency after gastric bypass: diagnosis, prevention and treatment. Diabetes Metab.

[73] Hammer HF (2012). Medical complications of bariatric surgery: focus on malabsortion and dumping syndrome. Digest Dis.

[74] Rubin DC, Levin MS (2016). Mechanisms of intestinal adaptation. Best Pract Res Clin Gastroenterol.

[75] de Luis DA, Pacheco D, Izaola O (2013). Micronutrient status in morbidly obese women before bariatric surgery. Surg Obes Rel Dis..

[76] Deitel M (2008). The change in the dumping syndrome concept. Obes Surg.

[77] Dykstra MA, Switzer NJ, Sherman V (2014). Roux en Y gastric bypass: how and why it fails?. Surg Curr Res.

[78] Bessler M, Daud A, Kim T (2007). Prospective randomized trial of banded versus non banded gastric bypass for the superobese: early results. Surg Obes Rel Dis.

[79] Rawlins ML, Teel D, Hedgcorth K (2011). Revision of Roux- en-Y gastric bypass to distal bypass for failed weight loss. Surg Obes Relat Dis.

[80] Karra E, Yousseif A, Batterham RL (2010). Mechanisms facilitating weight loss and resolution of type 2 diabetes following bariatric surgery. Trends Endocrin Met.

[81] Deitel M. Mini-gastric (one-anastomosis) bypass becoming a mainstream operation. *Bariatric News*, issue 18, 2013;13.

[82] Carbajo MA, Luque-de-Leon E (2016). Differentiating mini-gastric bypass/one-anastomosis gastric bypass from the single-anastomosis duodenoileal bypass procedures. Surg Obes Relat Dis.

[83] Carbajo MA, Luque-de-Leon E (2015). Mini-gastric bypass/one-anastomosis gastric bypass—standardizing the name. Obes Surg.

[84] Deitel M, Kular KS, Musella M, et al. A new organization—The MGB-OAGB Club. *Bariatric News* 2016;26:10. www.mgb-oagb-club.org; https://www.facebook.com/groups/mgboagbclub

